# Asymmetric Responses to Climate Change: Temperature Differentially Alters Herbivore Salivary Elicitor and Host Plant Responses to Herbivory

**DOI:** 10.1007/s10886-020-01201-6

**Published:** 2020-07-23

**Authors:** Sulav Paudel, Po-An Lin, Kelli Hoover, Gary W. Felton, Edwin G. Rajotte

**Affiliations:** Department of Entomology, The Pennsylvania State University, University Park, PA 16802 USA

**Keywords:** Assymetric responses, Global warming, Glucose oxidase, Insect-plant interactions, Induced plant defenses, Resistance, Salivary elicitors, Tolerance

## Abstract

The effect of temperature on insect-plant interactions in the face of changing climate is complex as the plant, its herbivores and their interactions are usually affected differentially leading to an asymmetry in response. Using experimental warming and a combination of biochemical and herbivory bioassays, the effects of elevated temperatures and herbivore damage (*Helicoverpa zea)* on resistance and tolerance traits of *Solanum lycopersicum* var. Better boy (tomato), as well as herbivory performance and salivary defense elicitors were examined. Insects and plants were differentially sensitive towards warming within the experimental temperature range. Herbivore growth rate increased with temperature, whereas plants growth as well as the ability to tolerate stress measured by photosynthesis recovery and regrowth ability were compromised at the highest temperature regime. In particular, temperature influenced the caterpillars’ capacity to induce plant defenses due to changes in the amount of a salivary defense elicitor, glucose oxidase (GOX). This was further complexed by the temperature effects on plant inducibility, which was significantly enhanced at an above-optimum temperature; this paralleled with an increased plants resistance to herbivory but significantly varied between previously damaged and undamaged leaves. Elevated temperatures produced asymmetry in species’ responses and changes in the relationship among species, indicating a more complicated response under a climate change scenario.

**Electronic supplementary material:**

The online version of this article (10.1007/s10886-020-01201-6) contains supplementary material, which is available to authorized users.

## Introduction

Consideration of asymmetry in plant-herbivore responses to climate warming is crucial to predicting how these systems will change over time. The global average temperature is predicted to rise by at least 4.0 °C by the end of the twenty-first century, resulting in increased frequency and intensity of drought and heat waves (Field 2014; Brown and Caldeira 2017). Rising temperatures can directly affect plant-herbivore relationships as the rates of insect metabolism and consumption are temperature-dependent. Plants also face challenges when exposed to multiple stresses (biotic and abiotic), and the plant’s response to mitigate one stressor may exacerbate another (Atkinson and Urwin 2012; Suzuki et al. 2014; Waterman et al. 2019). Previous predictions of insect pest populations and crop losses postulate an increase in both with elevated temperatures (Deutsch et al. 2018). However, the effects of climate change on plant-herbivore interactions may be asymmetric, with the plant, its herbivores and their interaction affected differentially. Additionally, the changes in one member in a plant-herbivore system can affect the response of the other leading to ecological impacts that are difficult to predict.

Insect-plant interactions in a warming climate will depend upon a range of independent and interactive factors such as sensitivity of insect and host plant, changes in host plant quality (chemistry, morphology and defense responses), and herbivore feeding behavior (compensatory or antagonistic). Being ectothermic, insect herbivores are directly influenced by temperature changes. Temperature increase within a range of critical thermal minimum (CTmin) and maximum (CTmax) accelerates insect metabolism leading to higher consumption and growth (Bale et al. 2002; Berggren et al. 2009). This changes the nutritional demands of insect herbivores (Lee et al. 2015). For example, protein denaturation increases at higher temperatures, which requires herbivores to consume protein-rich plants (Angilletta and Angilletta 2009). Similarly, efficiency of N digestion are also reduced in insect herbivores (e.g *Spodoptera exigua*) at elevated temperatures altering metabolic demands (Lemoine and Shantz 2016).

The changes in dietary requirements of insect affect the amount of caterpillar salivary elicitors (e.g Glucose Oxidase, GOX) (Peiffer and Felton 2005; Hu et al. 2008). Therefore, increasing temperatures alter herbivory (elicitor) derived plant defense responses (Rivera-Vega et al. 2017). Glucose oxidase (GOX), the most abundant salivary protein in *Helicoverpa zea* and commonly found in many lepidopteran larva, is secreted by the labial salivary glands during feeding and acts as an elicitor or suppressor of plant defenses, depending upon the host plant (Musser et al. 2002, 2005). Hydrogen peroxide (H_2_O_2_) is one of the enzymatic byproducts of GOX activity and acts as a secondary messenger that induces plant defenses in tomato (Orozco-Cárdenas et al. 2001; Tian et al. 2012).

Plant vegetative growth and reproductive success are strongly dependent on temperature. Each species in a particular environment has a specific or optimum temperature range for maximum productivity (Hatfield and Prueger 2015). For example, heat stress in most tomato varieties occurs at a mean temperature of 28–29 °C, a few degrees above the optimum range of 21–24 °C (reviewed by Hazra et al. 2007). Plant’s growth and reproductive success are compromised at above-optimum temperatures as photosynthetic ability is affected, resulting in limited energy reserves (Berggren et al. 2009; Sharkey and Zhang 2010; Sumesh et al. 2008; Todorov et al. 2003).

Temperature change induces phytochemical and morphological changes in host plants. In tomato (*S. lycopersicum* var. Heinz), the concentration of catecholic phenolics (chlorogenic acid and rutin) were significantly higher at a nighttime temperature of 17 °C than at other temperatures (Bradfield and Stamp 2004). Similarly, Rivero et al. (2003) reported a low level of polyphenol oxidase(PPO), peroxidase (POX) activity at 35 °C in tomato (*S. lycopersicum* cv Tmknvf_2_); Green and Ryan (1973) reported a significant reduction in the activity of protease inhibitors in tomato (*S. lycopersicum* var. Bonnie Best) at temperatures below 22 °C. Peroxidase activity in St. John’s wort (*Hypericum perforatum* cv. Topas) was, in contrast, increased at elevated temperatures (Zobayed et al. 2005). The density of leaf trichomes typically increases at elevated temperatures (Ehleringer and Mooney 1978; Pérez-Estrada et al. 2000; Bickford 2016). Limited information, however, exists on the combined effect of elevated temperature and insect herbivores on induction of plant defenses (Bidart-Bouzat and Imeh-Nathaniel 2008).

Studies on warming-induced changes in host plant quality and subsequent effects on herbivore performance have produced mixed results (Jamieson et al. 2017; Zavala et al. 2008; Zvereva and Kozlov 2006). In alfalfa (*Medicago sativa*), concentrations of plant secondary metabolites (sapogenins and saponins) were increased at higher temperatures, depressing caterpillar growth (*Spodoptera exigua*). In contrast, the Green-veined butterfly (*Pieris napi*) responded to warming-mediated poor-quality foliage in Brassicaceae by consuming a significantly higher amount of plant tissue (Bauerfeind and Fischer 2013). The performance of aphids (*Myzus persicae* and *Brevicoryne brassicae*), however, was not affected when fed on oilseed rape plants with differences in nutritional quality exposed to different temperatures (Himanen et al. 2008). Furthermore, temperature induced changes on tobacco (*Nicotiana tabacum*) and devil’s claw (*Proboscidea louisianica*) were so impactful to the tobacco hornworm (*Manduca sexta*) that it reversed the widely accepted temperature-size rule, which predicts an increased final mass of ectotherms (e.g insects) at low temperatures (Diamond and Kingsolver 2010).

Plants have acquired tolerance strategies, which may be affected by warmer temperatures. Tolerance minimizes plant fitness costs in response to herbivores, which can be manifested in physiological and developmental traits such as altered photosynthetic ability and regrowth capacity (Bita and Gerats 2013; Mitchell et al. 2016). Photosynthetic activity was reduced in wild parsnip (*Pastinaca sativa*) following herbivore damage (Zangerl et al. 2002). However, evidence of compensatory photosynthesis in response to defoliation (e.g. in *Salix planifolia ssp. planifolia*; Houle and Simard 1996) has been noted, but it is not universal. Regrowth capacity or compensatory growth is an adaptive mechanism to stimulate growth in response to biotic and abiotic stresses (e.g. temperature) (Agrawal 2007; Bjorndal et al. 2003; Gong et al. 2015; Liu et al. 2012). Han et al. (2015) reported a reduction in compensatory growth with high-temperature stress. The combined effect of temperature and herbivory on plant tolerance traits are largely unknown (Jamieson et al. 2012).

It is time to dissect the broad predictions of biological population changes under climate warming and delve into the details of how individual species might change and how the interaction of these changes among species might produce new relationships that are not simply linear projections of individual species change. Consideration of this asymmetric change will give a truer picture of what is to come. The purpose of this study was to examine potential asymmetric responses of elevated temperatures on tomato (*Solanum lycopersicum* var. Better boy) in the presence of an herbivore, *Helicoverpa zea* that uses this crop as one of its major host plants. First, we hypothesized that elevated temperature affects performance of insect herbivore and host plant differently resulting in an asymmetry. Second, level of salivary defense elicitors in caterpillars is temperature-dependent subsequently affecting plant defense responses. Third, plant- and herbivory (elicitor)-derived changes in host plants in response to elevated temperatures will affect herbivore growth influencing overall insect-plant interactions. In plant, we measured changes in plant growth and leaf phytochemical and morphological traits (leaf defensive proteins; trypsin protease inhibitors (TPI) and polyphenol oxidase (PPO), and leaf trichomes). For the herbivore, we measured larval growth and development as well as activity of the caterpillar salivary defense elicitor, glucose oxidase (GOX). For the interactions between plant and herbivore at different temperatures we compared herbivore growth on damaged and undamaged plants, induction of plant defenses, and tolerance capacity (insect feeding damage recovery and shoot regrowth ability).

## Materials and Methods

### Temperature Treatments

Growth chambers (Caron, 700X/730X-50/75-X Series) at Pennsylvania State University, University Park, PA during 2017–2018 were used for both insects and plants and allocated to three different day/night temperature treatments- i) 25 °C/14 °C (ambient temperature; T_A,_ mean = 19.5 °C), based on the mean temperature in the Mid-Atlantic United States (40.7934° N, 77.8600° W) during the normal tomato growing season (statecollege.com, 2018), ii) 30 °C/18 °C (elevated temperature 1; T_E1_), and iii) 35 °C/ 22 °C (elevated temperature 2; T_E2_). The T_E1_ and T_E2_ are 4.5 °C above T_A_ and 4.5 °C above T_E1_ respectively, consistent with the temperature increase expected by the end of this century (Brown and Caldeira 2017). Additionally, T_A_ falls in the below-optimum range for normal tomato growth and production, T_E1_ is within the optimum range, and T_E2_ is above-optimum (Hazra et al. 2007). For the insect herbivore (*H. zea*), the maximum and minimum developmental temperature threshold for larvae are 36 °C and 12.5 °C, respectively (Mangat and Apple 1966; Butler 1976). Thus, T_E2_ is close to the thermal limit for *H. zea*, whereas T_A_ and T_E1_ are within the maximum and minimum threshold range. The experiments were conducted in three temperoral blocks and seedlings were randomly allocated to temperature treatments. Chambers were switched for each block to ensure that the observerved effects were not due to any differences between the chambers.Temperatures inside growth chambers were monitored constantly with a digital thermometer.

### Effect of Temperature on Insect Herbivore

*Helicoverpa zea* eggs were obtained from Benzon Research (Carlisle, PA, USA). *H. zea* (Family: Noctuidae) is a generalist herbivore, also known as corn earworm or tomato fruit worm, and is a major agricultural pest of a wide variety of crops including tomato (Fitt 1989). For majority of the experiments, neonates were reared individually inside a plastic cup until the end on a wheat gern and casein-based artificial diet (30 ml) (Peiffer and Felton 2005). However, neonates were fed on the tomato leaves for measuring relative growth rates on leaf samples.

#### Growth and Development

The effect of temperature on larval growth rate, larval duration, pupal duration and pupal mass were evaluated at T_A_, T_E1_ and T_E2_, using 60–70% RH and a photoperiod of 16:8 L:D. Mean larval weights (g) (*n* = 81–83) were recorded after 5 days. Times to reach pupation from neonate (days) (Larval period) (*n* = 60–62) and from pupation to adult (days) (pupation period) (n = 60–62) were also recorded. Pupal weights (g) (n = 60–62) were measured 48 h after entering the pupal stage. To ensure high moisture content in the artificial diet, the diet was replaced every two days.

#### Glucose Oxidase (GOX) Enzyme Assay and Protein Determinations

To evaluate if temperature affects the caterpillar defense elicitor, GOX (enzyme activity and protein amount), *H. zea* salivary glands (from T_A_ and T_E2_) were dissected from actively feeding 5th instars (*n* = 26–29/treatment) (Tian et al. 2012). Glands were homogenized with phosphate buffer (0.1 M, pH 7) and supernatant was collected after centrifugation (4 °C, 7500×*g*, 10 min) (Eichenseer et al. 1999). The GOX enzyme activity was quantified using a spectrometer at the temperature at which larvae were reared- 25 °C for T_A_-samples and 35 °C for T_E2_-samples. Homogenized samples were also used to extract GOX proteins with sodium dodecyl sulfate polyacrylamide gel electrophoresis (SDS-PAGE) (*n* = 4). Western blots were blocked using 1:10,000 diluted anti-GOX antibody (Peiffer and Felton 2005). Band intensity on the Western blot gel was quantified using image analysis software (Adobe Photoshop CC 2018 (version 19)). RNA extraction and cDNA synthesis were also conducted as described by Tan et al. (2018). The *gox* gene expression was tested by qRT-PCR analysis using actin (ACT) as a reference gene. Relative gene expression was calculated using the 2^−ΔΔct^ method (*n* = 5–6) (Livak and Schmittgen 2001).

### Effect of Temperature on Host Plants

Tomato (*Solanum lycopersicum* cv Better Boy) seeds were procured commercially (Harris seeds, Rochester, NY, USA). Seedlings were grown in Metromix 400 potting mix (Premier Horticulture, Quakertown, PA, USA) in growth chambers (T_A_, T_E1_ and T_E2_) with a 16 L:8D h photoperiod during 2017–18 until the end of the experiment. Relative humidity (RH) was maintained at 60–70% with photosynthetic active radiation (PAR) of 300 μmol m^−2^ s^−1^. A continuous supply of water was provided (every 1–2 days) to ensure that the plants were not water-stressed.

#### Growth and Development

Roots and shoots of 3-week old plants (*n* = 8) were separately removed and dried in an oven (60 °C for 48 h) to compare the dry weight (DW) of shoot and root biomass for the three different temperature treatments (T_A_, T_E1_ and T_E2_).

#### Plant Defense Responses

The activities of two jasmonic acid (JA)-related defensive proteins, PPO and TPI, were measured as a proxy for host plant defense responses against *H. zea*. PPO and TPI play important roles in enhancing plant defenses in tomato, particularly against *H. zea* (Broadway and Duffey 1986; Felton et al. 1989; Bhonwong et al. 2009). Two different experiments were conducted to measure the plant-derived and herbivory-derived effects of temperature on plant defense responses.

#### Plant Derived Effects

Tomato seedlings were grown at three different temperature regimes, whereas *H. zea* larvae were reared on artificial diet in a common incubator at a constant day/night temperature (T_C_: 23 °C/19 °C) until placed on experimental plants. At the four-leaf stage, fully expanded terminal leaflets (with and without caterpillar damage) were used as the focal leaves for defensive protein bioassays (Tan et al. 2018). Leaflets were damaged by allowing 5th instar *H. zea* to completely feed (usually 2–3 h) on leaf tissues inside a clip cage (3.15 cm^2^), while an empty clip cage was used for the ‘control’ leaflets (terminal). Leaf tissues were sampled from the local leaves. PPO and TPI activities were measured and compared after 48 h of caterpillar damage using a spectrophotometric method (Acevedo et al. 2017). PPO activity was expressed as mOD/min/mg protein; TPI activity was first calculated as TPI (%) = (1-(slope of sample/slope of noninhibitor)) × 100 and then normalized by the protein amount (mg) in the sample (% inhibition/mg protein).

#### Herbivory (elicitor)-derived effects

*H. zea* caterpillars were reared on artificial diet under two different day/night temperature regimes, T_A_ (25 °C/14 °C) and T_E2_ (35 °C/22 °C). Fifth instar larvae were placed on fully expanded terminal leaflets from four-week old tomato plants (*n* = 10–13) that were grown at a constant temperature and allowed to feed on leaf tissues inside a clip cage (3.15 cm^2^) under greenhouse conditions (temperature: 27 °C ± 2 °C, humidity: 60–70%, 16 h daylight). PPO and TPI activities were analyzed after 48 h of caterpillar damage as described above to evaluate temperature effects on the ability of herbivory to induce plant defenses. Further, an excised leaf bioassay with 1st instar larvae was conducted for 24 h to test if herbivory (elicitor)-derived changes in plant defense responses influence growth rate of herbivory. Two-days before the bioassay experiment, terminal leaflets from four-week old tomato plants were first mechanically wounded followed by application of 15 μL of salivary gland supernatant from two caterpillar treatments (T_A_ (25 °C/14 °C) and T_E2_ (35 °C/22 °C)). Supernatant were collected from each caterpillar treatment as described above and was diluted to 1 μg/μL using Bradford assay (Bradford 1976). The ‘control’ leaves were mechanically wounded but did not receive gland supernatant. First-instar *H. zea* were then allowed to feed on excised leaves from two caterpillar treatments for 24 h and the relative growth rate (RGR) was calculated as:

$$ \mathrm{RGR}\ \left(\mathrm{weight}\ \mathrm{gain}/\mathrm{g}/\mathrm{day}\right)=\left({\mathrm{W}}_1-{\mathrm{W}}_0\right)/\Big(\left({\mathrm{d}}_1-{\mathrm{d}}_{0\Big)}\ast {\mathrm{W}}_0\right), $$

Where, W_1_ and W_0_ are larval weight at days, d_0_ and d_1_, and W_0_ is the initial larval weight before the start of the experiment (Waldbauer 1968).

#### Density of Leaf Trichomes

Fourteen days post caterpillar feeding, the youngest terminal leaflets were randomly selected from plants (*n* = 10) from each temperature treatment to compare the density of trichomes on the adaxial leaf surface (Paudel et al. 2019). Both glandular and non-glandular trichomes were counted. Two leaf discs of 0.6-cm diameter were punched out from each side of the mid-vein of a leaflet, and the density (number/cm^2^) of all glandular and non-glandular trichomes was determined using a light microscope.

### Effect of Temperature on Insect-Plant Interactions

Two different strategies, resistance and tolerance, were measured as determinants of insect-plant interactions (Mitchell et al. 2016). Herbivore feeding bioassays were used to measure the plants’ resistance, whereas compensatory photosynthesis and regrowth ability were examined to measure plant tolerance.

#### Herbivore Feeding Bioassay

Excised leaf bioassays with damaged (D) or undamaged (UD) leaves from three different temperature regimes (T_A_, T_E1_ and T_E2_) were used to measure the temperature effect on host plant quality and resistance to herbivores. The full expanded terminal leaflet from a four-week old tomato plant was damaged by allowing a single 5th instar *H. zea* to feed inside a clip cage (3.15 cm^2^), whereas an empty cage was placed on undamaged leaves (control). At 48 h post-damage, randomly selected 1st instars (*n* = 30) from a stock colony were individually weighed (day 0) and placed into plastic cups (30 ml) with ‘damaged’ and ‘undamaged’ leaves from plants grown under T_A_, T_E1_ and T_E2_-treatments. Individual larvae were then weighed after 48 h and the relative growth rate (RGR) was calculated as described above.

#### Photosynthesis Rate

Three-week old plants (*n* = 11) were randomly selected from a group of plants grown in growth chambers under three temperature regimes (T_A_, T_E1_ and T_E2_). The rate of photosynthesis (μmol m^−2^ s^−1^) in both damaged and undamaged leaves was repeatedly measured at three different time points, 2 h, 24 h and 120 h post-damage. Fully expanded terminal leaflets were damaged similarly by *H. zea* larvae as described above. The distal portion of each terminal leaflet (6-cm^2^ leaf area) was inserted into a cuvette connected to a Li-Cor 6400 (Li-C0r, Lincoln, NE) gas-exchange system with a red/blue LED light source (irradiance of 600 μmol m^−2^ s^−1^_)_. The CO_2_ concentration of the incoming air was adjusted to 400 μmol at a flow rate of 200 μmol m^−2^ s^−1^. Output CO_2_ was measured to determine the rate of photosynthesis as μmol m^−2^ s^−1^ (Meyer and Whitlow 1992).

#### Regrowth Ability (Compensatory Growth)

The shoot tissue above the 2nd mature leaf from 3-week old plants (*n* = 12) grown under three temperature regimes (T_A_, T_E1_ and T_E2_) were removed mechanically to simulate herbivore (Moreira et al. 2012; Dostálek et al. 2016). Biomass of tissues removed were determined as dry weight (DW; g, 70 °C for 72 h in oven). Plants were then placed back inside respective growth chambers. After 10 days, the shoot regrowth above the 2nd mature leaf was again removed and dry mass was determined. Regrowth percent (%) was calculated as (Van Der Meijden et al. 2000):

$$ \mathrm{Regrowth}\ \mathrm{percent}\ \left(\%\right)=\left(\mathrm{dry}\ \mathrm{weight}\ \left(\mathrm{g}\right)\ \mathrm{of}\ \mathrm{emerged}\ \mathrm{shoot}/\mathrm{biomass}\ \mathrm{of}\ \mathrm{the}\ \mathrm{shoot}\ \mathrm{removed}\ \mathrm{earlier}\ \left(\mathrm{DW};\mathrm{g}\right)\right)\%. $$

### Statistical Analyses

Using a completely randomized block design, larval weight gain (g/day), pupal mass (g), developmental time (larval and pupal period in days), GOX enzyme activity, GOX protein determinations (band intensity), *gox* gene expression, activities of defensive proteins (herbivory-mediated effect), root and shoot biomass (g), and herbivore RGR were analyzed using one-way ANOVA with temperature as the main effect and block as the random effect. Experiments on plant defensive proteins activities (plant-derived effect), trichome density, and herbivore RGR were analyzed using a two-way ANOVA with the main effects being temperature and insect treatment (damaged or undamaged) plus all interaction terms and block as the random effect. Photosynthetic rates (determined at multiple time points) were analyzed with a repeated-measures ANOVA using temperature and treatment (damaged or undamaged leaflets) as independent variables. Generalized linear model with logistic distribution was used to anaylize shoot regrowth (%) data. Means were separated with Tukey’s Honest Significant Differences (HSD) mean comparison tests. Data were checked for normality and analyzed using ‘Minitab 18.0’ software (Minitab Inc. 2018).

## Results

### Effect of Temperature on Insect Herbivore

#### Herbivore Growth and Development

**Fig. 1:**
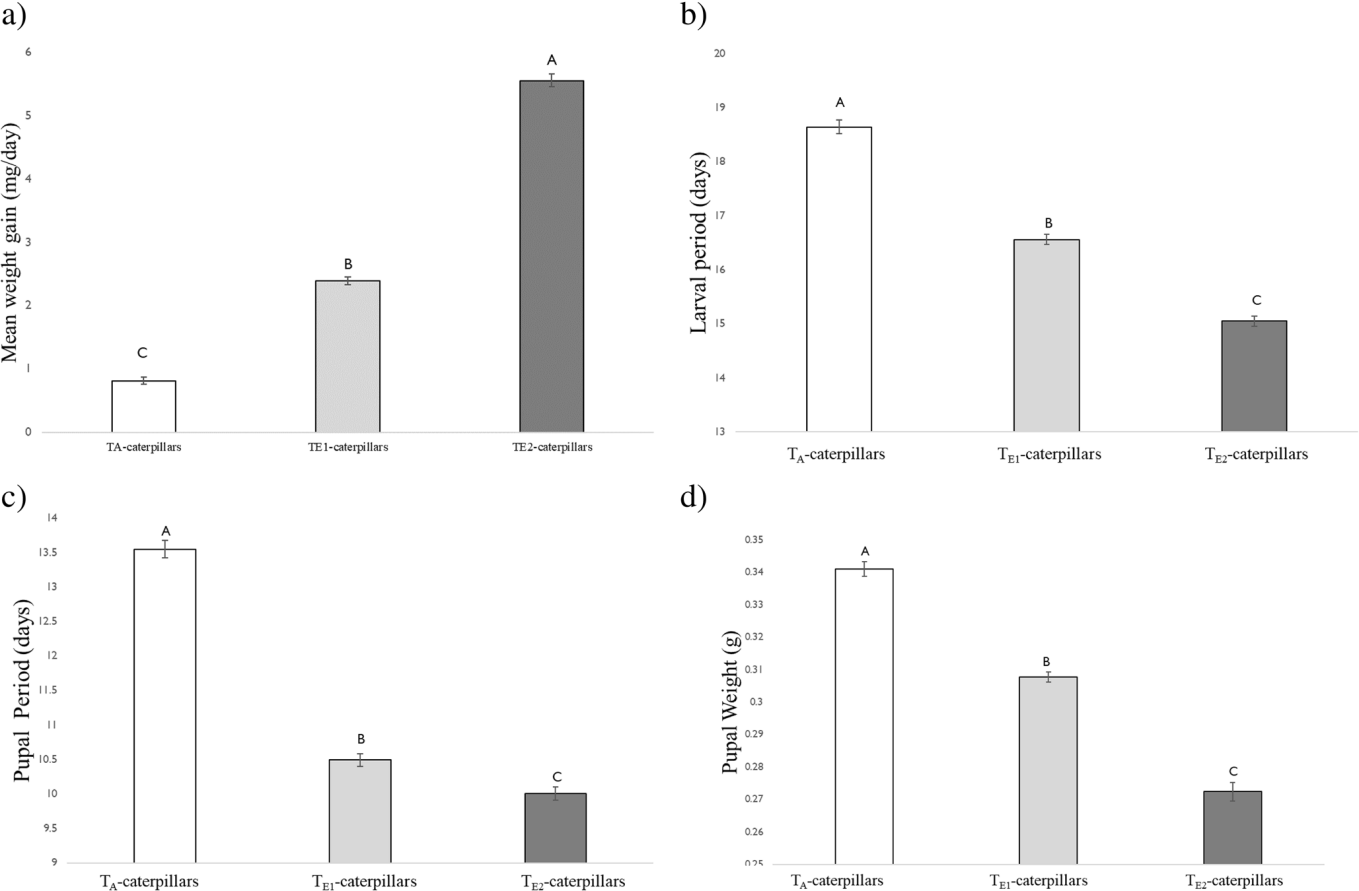
Growth and development of *H. zea* reared on artificial diets at three different day/night temperatures: 25 °C/14 °C (ambient temperature; T_A_-caterpillars), 30 °C/18 °C (elevated temperature 1; T_E1_-caterpillars) and 35 °C/ 22 °C (elevated temperature 2; T_E2_-caterpillars). a) Mean larval weight gain (g/day) calculated by dividing the weight of the larvae after 5 d by the number of days (n) b) Larval period calculated by counting number of days (n) from neonate to pupa c) Pupal period calculated by counting number of days (n) from pupation to adult, and d) Mean pupal weight (g). Bars are mean ± SEM and means with different letters are statistically different as determined by a Tukey HSD. There was significant effect of temperature on larval weight gain (F = 1003, df = 2, *P* < 0.001), larval period (F = 250.1, df = 2, P < 0.001), pupal period (F = 345.5, df = 2, P < 0.001), and pupal weight gain (F = 230.84, df = 2, P < 0.001)

There was a significant effect of temperature on *H. zea* growth when they fed on artificial diet (Fig. 1a). Larval growth increased with elevated temperatures; growth at T_E2_ (35 °C/ 22 °C) was 2.3-fold and 6.8-fold higher compared to T_E1_ and T_A_, respectively. Both the larval and pupal durations were significantly shorter at the highest temperature regime, T_E2_ (Fig. 1b and c). On average, T_E2_- caterpillars took 15.05 d and 10 d to complete their larval and pupal stages, whereas T_A_- caterpillars took 18.6 and 13.5 d, respectively. Pupal weight was significantly lower at T_E2_ compared to both T_E1_ and T_A_ (Fig. 1d).

#### Glucose Oxidase (GOX) Enzyme Assay and Protein Determinations

**Fig. 2:**
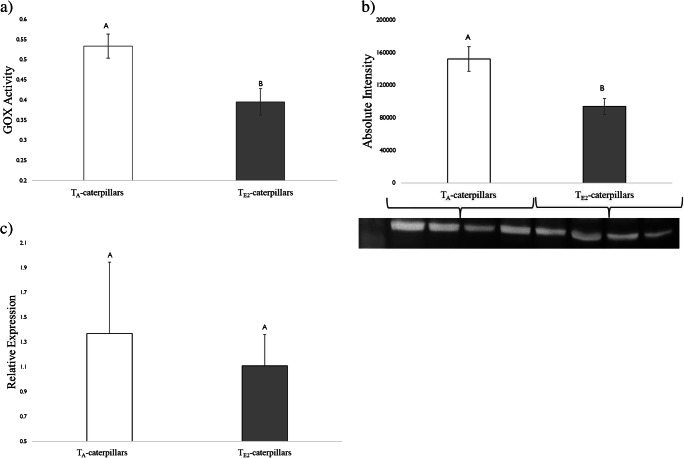
Glucose oxidase (GOX) enzyme activity and transcript levels in labial salivary glands of 5th instar *H. zea* reared at two different day/night temperatures: 25 °C/14 °C (ambient temperature; T_A_-caterpillars) and 35 °C/ 22 °C (elevated temperature 2; T_E2_-caterpillars). a) GOX enzyme activity (mOD/min/mg protein) b) Absolute intensity of bands from immunoblot analysis of glucose oxidase (GOX) protein, and c) Relative expression of gox transcript levels. Caterpillars were reared on artificial diet post-hatching. Bars are mean ± SEM and means with different letters are statistically different as determined by a Tukey HSD. There was a significant effect of temperature on GOX enzyme activity (F = 8.5, df = 1, P < 0.001) and band intensity (F = 8.0, df = 1, *P* < 0.05), but not on *H. zea gox* transcript level (F = 0.7, df = 1, *P* = 0.15)

Temperature had a significant effect on the activity of GOX in the labial salivary glands of *H. zea* caterpillars (Fig. 2a)*.* T_A_-caterpillars had 1.2-fold higher GOX activity than the T_E2_-treated caterpillars. This result was further confirmed by the immunoblot analyses, where a reduction in GOX protein accumulation in the labial glands of larvae reared at T_E2_ compared to T_A_ was observed (Fig. 2b). However, there was no significant difference in the transcript level of gene encoding *gox* (Fig. 2c).

### Effect of Temperature on Host Plants

#### Growth and Development

**Fig. 3:**
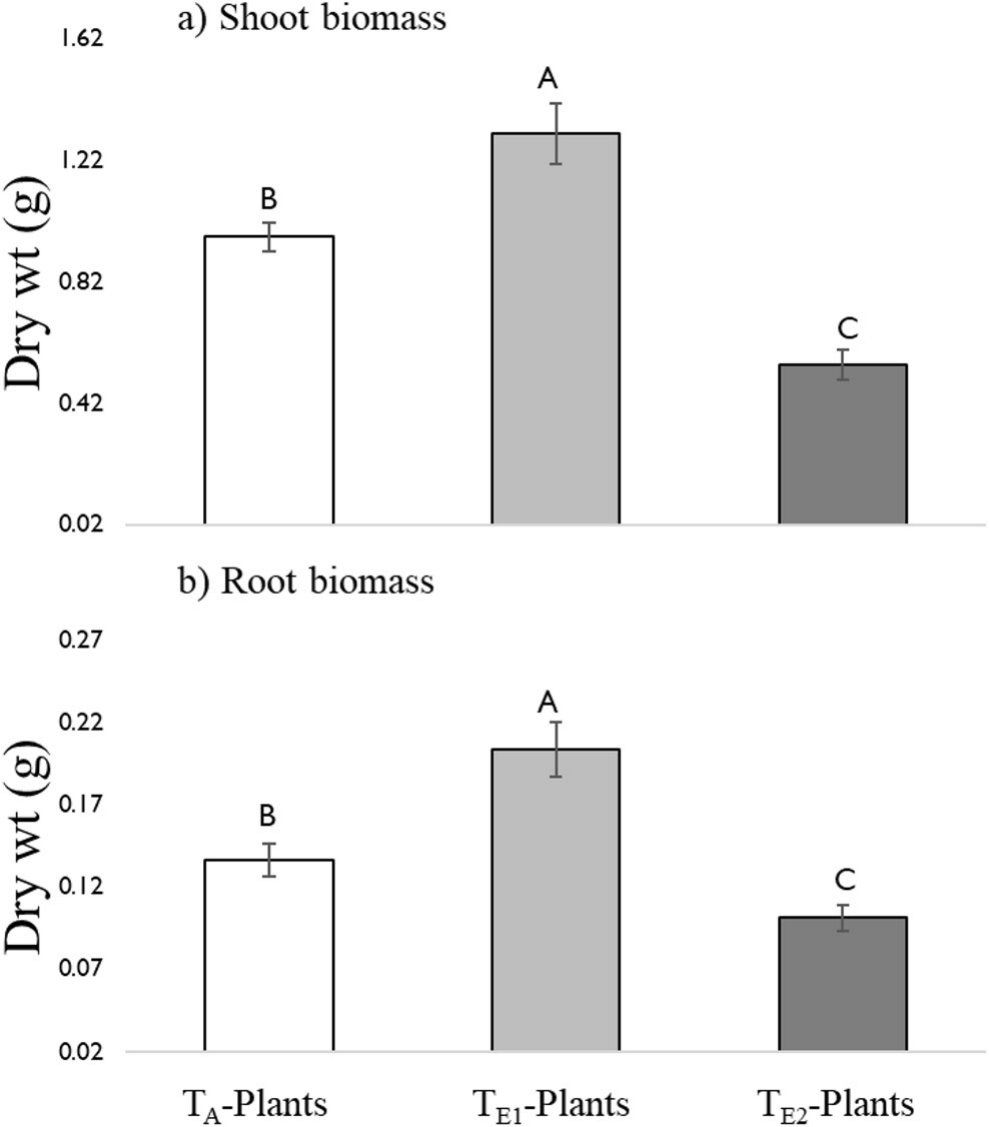
Growth and development of tomato plants, a) Shoot biomass (dry weight (g)) and b) root biomass (dry weight (g)) of three-week old plants grown at three different day/night temperatures: 25 °C/14 °C (ambient temperature; T_A_-plants), 30 °C/18 °C (elevated temperature 1; T_E1_-plants) and 35 °C/ 22 °C (elevated temperature 2; T_E2_-plants). Bars are mean ± SEM and means with different letters are statistically different as determined by a Tukey HSD. Temperature had a significant effect on both shoot (F = 209.7, df = 2, *P* < 0.001) and root biomass (F = 131.7, df = 2, P < 0.001)

Temperature had a significant effect on both shoot and root biomass of tomato plants (Fig. 3a and b). With elevated temperature (T_E1_), root and shoot biomass was increased but declined with T_E2_. On average, T_E1-_plants had 1.3-fold and 2.4-fold higher shoot biomass, and 1.5-fold and 2-fold higher root biomass than those grown at T_A_ and T_E2_, respectively.

### Plant Defense Responses

#### Plant-Derived Effects

##### Trypsin Protease Inhibitor

**Fig. 4:**
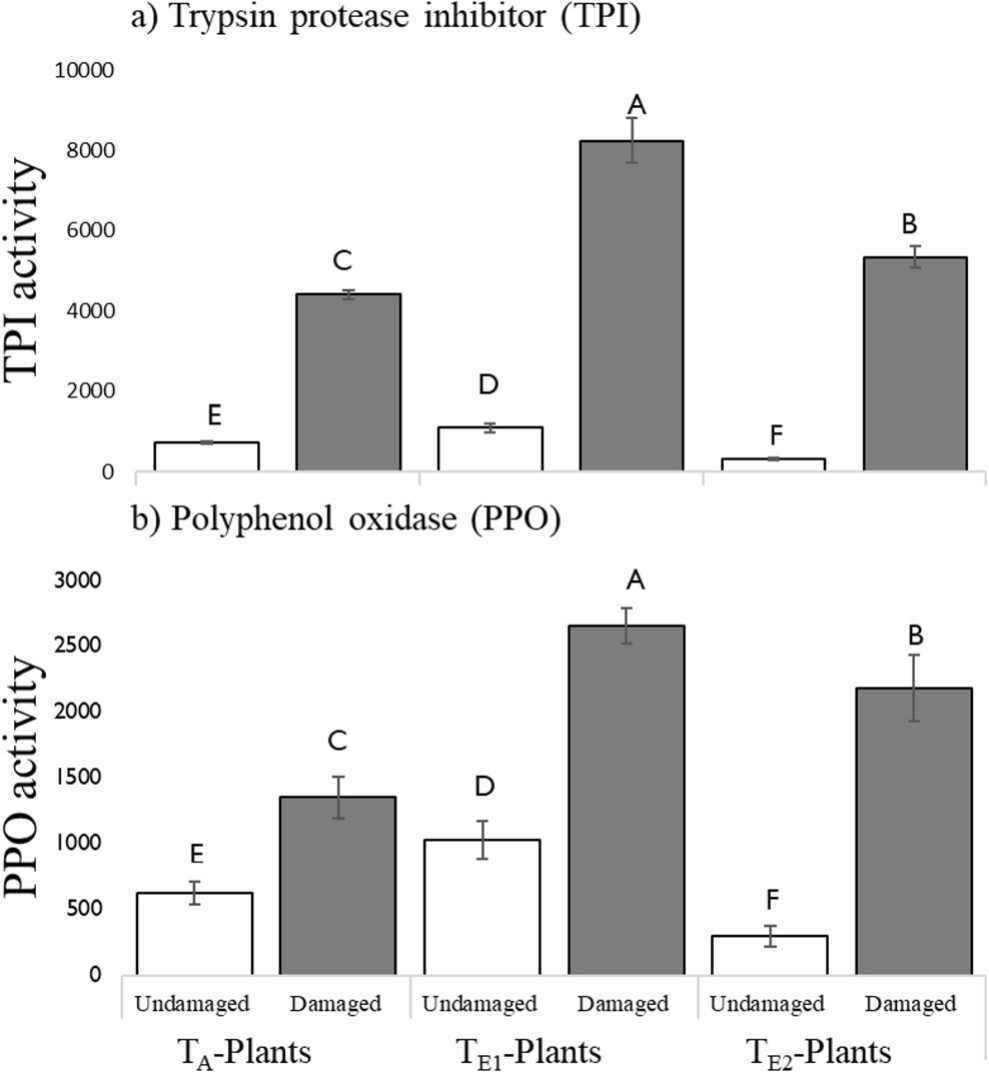
Activity of defensive proteins: a) Trypsin protease inhibitor (TPI; mOD/min/mg protein) and b) Polyphenol oxidase (PPO; % inhibition/mg protein) activity in undamaged and damaged leaves from four-leaf stage plants grown at three different day/night temperature: 25 °C/14 °C (ambient temperature; T_A_-plants), 30 °C/18 °C (elevated temperature 1; T_E1_-plants) and 35 °C/ 22 °C (elevated temperature 2; T_E2_-plants). Fifth-instar *H. zea,* reared at a common day/night temperature (T_C_: 23 °C/19 °C) were used to damage leaves. Bars are mean ± SEM and means with different letters are statistically different as determined by a Tukey HSD, *P* < 0.05. There were significant temperature (PPO; F = 147.6, df = 2, P < 0.001,TPI: F = 357.5, df = 2, P < 0.001), insect damage (PPO: F = 1150.6, df = 1, *P* < 0.001, TPI: F = 5741.3, df = 1, *P* < 0.001), and interactive effects of temperature and insect damage (PPO: F = 70.8, df = 2, *P* < 0.001, TPI: F = 209.4, df = 2, P < 0.001) on PPO and TPI activities

Activity of TPI in leaves was significantly affected by both temperature and insect damage (Fig. 4a). In both undamaged and damaged leaves, the TPI activity in T_E1_-plants was highest. While T_E2_-plants had the lowest activity of TPI in undamaged leaves, they had the highest percent (%) induction (17-fold increase) followed byT_E1_ (8.5-fold increase) and T_A_ (7-fold increase) in response to caterpillar damage.

##### Polyphenol Oxidase (PPO)

There were significant effects of temperature and insect feeding on PPO activity (Fig. 4b). Compared to both T_A_ and T_E2_, plants grown at T_E1_ had significantly higher PPO activity in both damaged and undamaged leaves. In contrast, percent (%) induction of PPO following larval damage was highest at T_E2_ (3.5-fold increase), followed by T_A_ (2.5-fold increase) and T_E1_ (2.3-fold increase).

### Herbivory (Elicitor)-Derived Effects

**Fig. 5:**
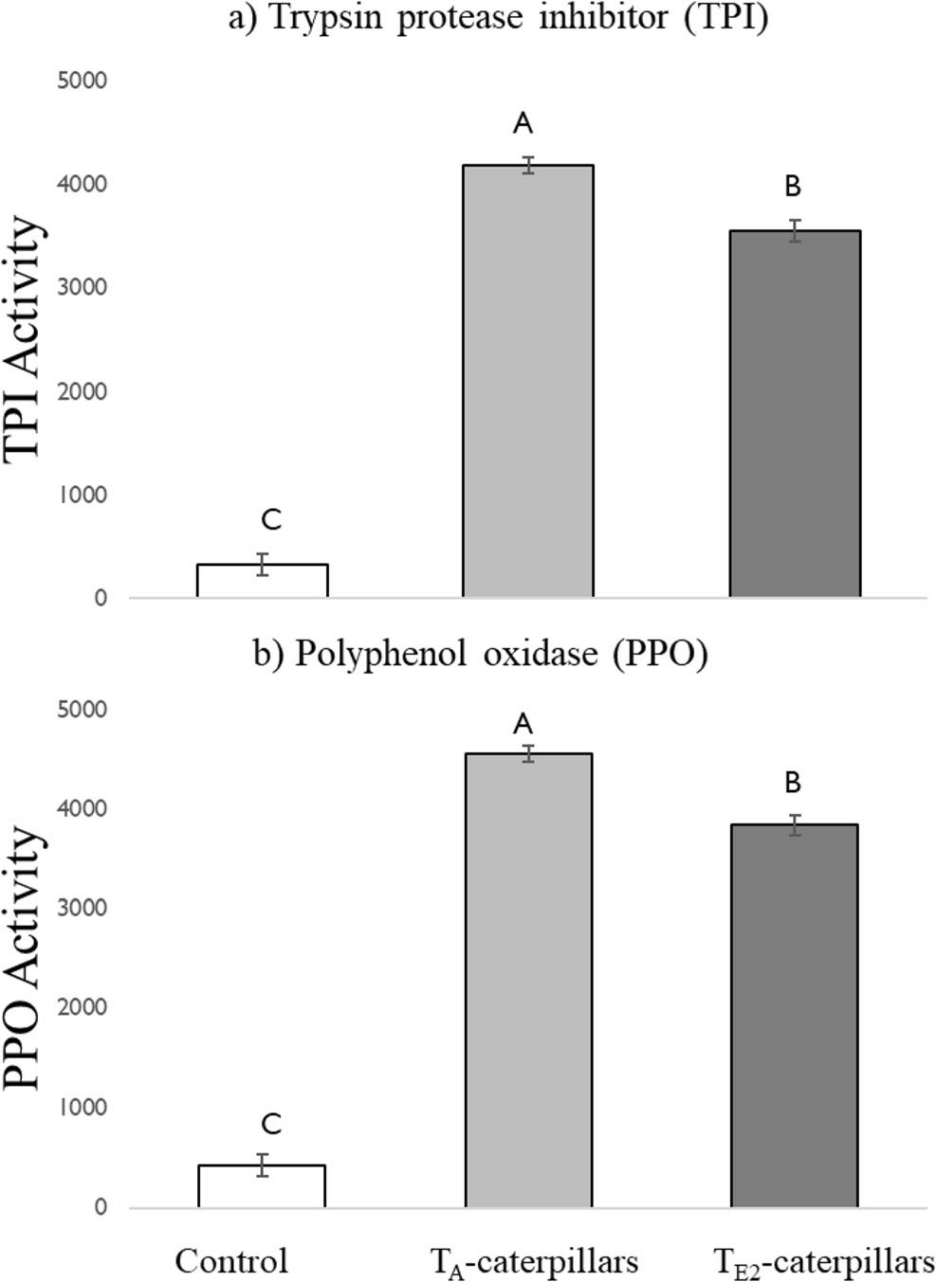
Activity of defensive proteins: a) Trypsin protease inhibitor (TPI; mOD/min/mg protein) and b) Polyphenol oxidase (PPO; % inhibition/mg protein) activity in leaves damaged by 5th instar *H. zea*, which were reared at two different temperature regimes: 25 °C/14 °C (ambient temperature; T_A_-caterpillars) and 35 °C/ 22 °C (elevated temperature 2; T_E2_-caterpillars). Control plants didn’t receive any herbivore treatment. Plants were grown in a common greenhouse environment. Caterpillars were reared on artificial diet until placed on experimental leaves. Bars are mean ± SEM and means with different letters are statistically different as determined by a Tukey HSD. There was a significant effect of caterpillar rearing temperature on PPO (F = 169.9, df = 1, *P* < 0.001) and TPI (F = 153.1, df = 1, *P* < 0.001) activities

When fed on leaves from plants grown at a common temperature (T_C_), caterpillars reared under a warmer temperature (T_E2_) induced significantly lower levels of both TPI and PPO activities in plants (Fig. 5a and b). On average, the activity of PPO and TPI was 1.1-fold and 1.6-fold higher, respectively, in the leaves damaged by T_A_-caterpillars compared to T_E2_-caterpillars.

**Fig. 6:**
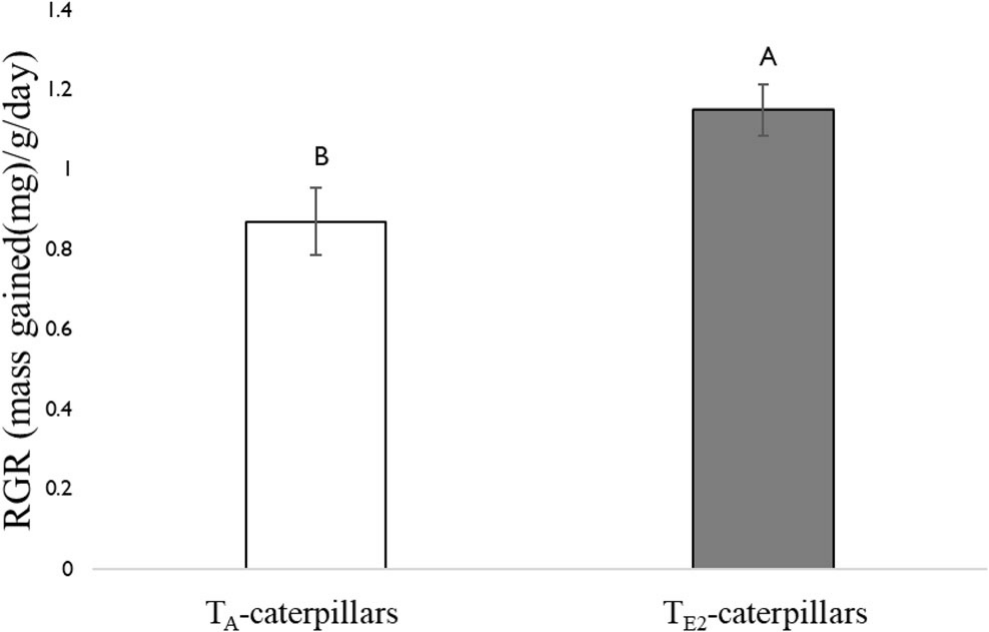
Relative growth rate (RGR) (mass gained((mg)/g/day)) of 1st instar *H. zea* fed on detached leaves to which salivary gland homogenate was added from 5th instar *H. zea* reared at two different temperature regimes: 25 °C/14 °C (ambient temperature; T_A_-caterpillars) and 35 °C/ 22 °C (elevated temperature 2; T_E2_-caterpillars). Gland homogenate was applied 2 d prior to collecting leaves for bioassays. Tomato plants were grown in a common greenhouse environment. Caterpillars were reared on artificial diet. Bars are mean ± SEM and means with different letters are statistically different as determined by a Tukey HSD (F = 6.6, df = 1, P < 0.05)

In addition, the growth rate of caterpillars was significantly higher when fed on leaves treated with salivary gland homogenate from T_E2_-caterpillars compared to those from T_A_-caterpillars (Fig. 6).

#### Density of Leaf Trichomes

**Fig. 7:**
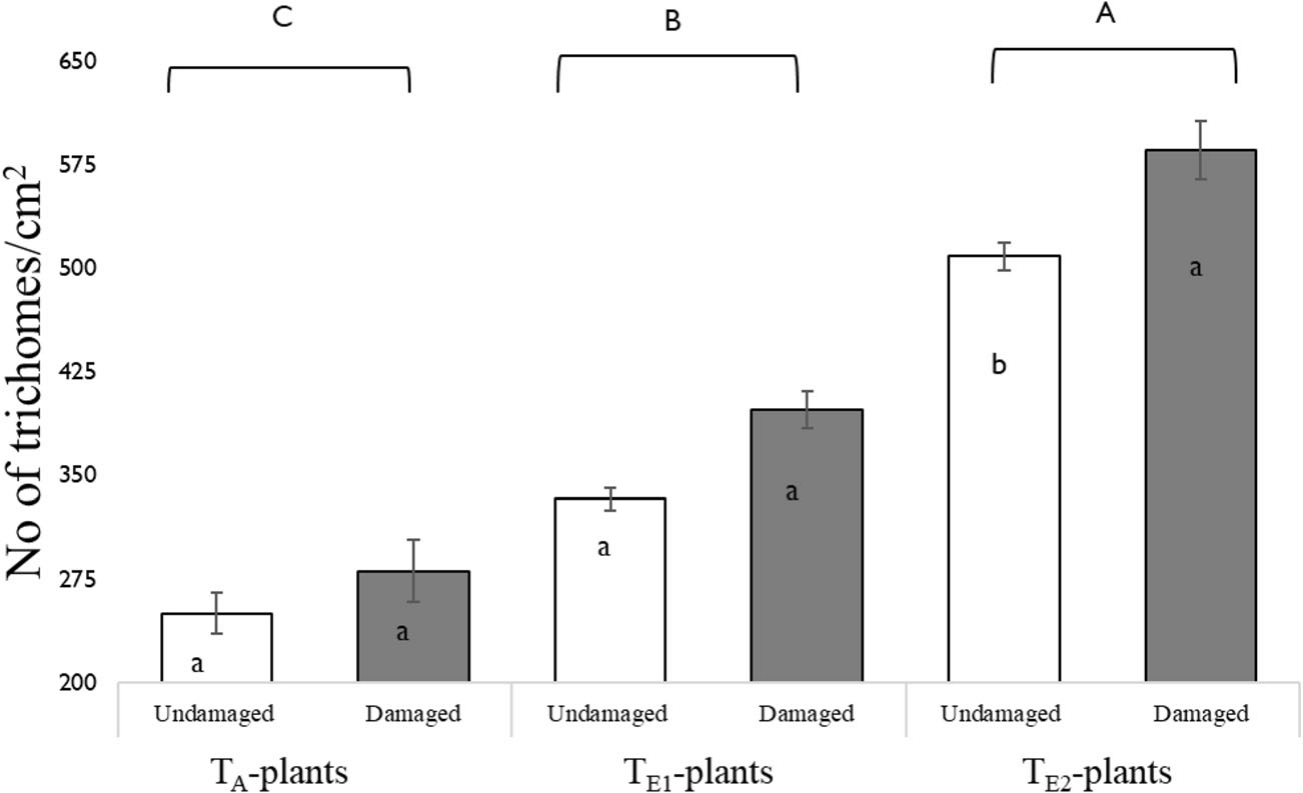
Density of glandular and non-glandular trichomes (number of trichomes/cm^2^) on undamaged (UD) and damaged (D*)* leaf surface (adaxial) at three different day/night temperatures: 25 °C/14 °C (ambient temperature; T_A_-plants), 30 °C/18 °C (elevated temperature 1; T_E1_-plants) and 35 °C/ 22 °C (elevated temperature 2; T_E2_-plants). Fifth-instar *H. zea,* reared at a common day/night temperature (T_C_: 23 °C/19 °C) were used to damage leaves; trichomes on damaged and undamaged leaves were counted 14 d post-damage. Bars are mean ± SEM and means with different letters are statistically different as determined by a Tukey HSD. Differences are between ‘damaged’ and ‘undamaged’ leaves within temperature. Both temperature (F = 159.2, df = 2, *P* < 0.001) and insect damage (F = 19.3, df = 1, P < 0.001) had significant effects on the density of leaf trichomes. There was no interactive effect of temperature and insect damage (F = 1.15, df = 2, *P* = 0.324)

Temperature and insect damage each had a significant effect on leaf trichome densities, but there was no significant interaction (Fig. 7). In both damaged and undamaged leaves, the density of leaf trichomes increased at warmer temperatures. On average, T_E2_-plants had a 1.5 and 2-fold higher trichome density in undamaged leaves and and a 1.47 and 1.41-fold higher trichome density in damaged leaves compared to T_E1_ and T_A_-plants respectively. Post-insect damage, a significant induction of trichomes was only noted in T_E2_-plants.

### Effect of Temperature on Insect-Plant Interactions

#### Herbivore Feeding Bioassay

**Fig. 8:**
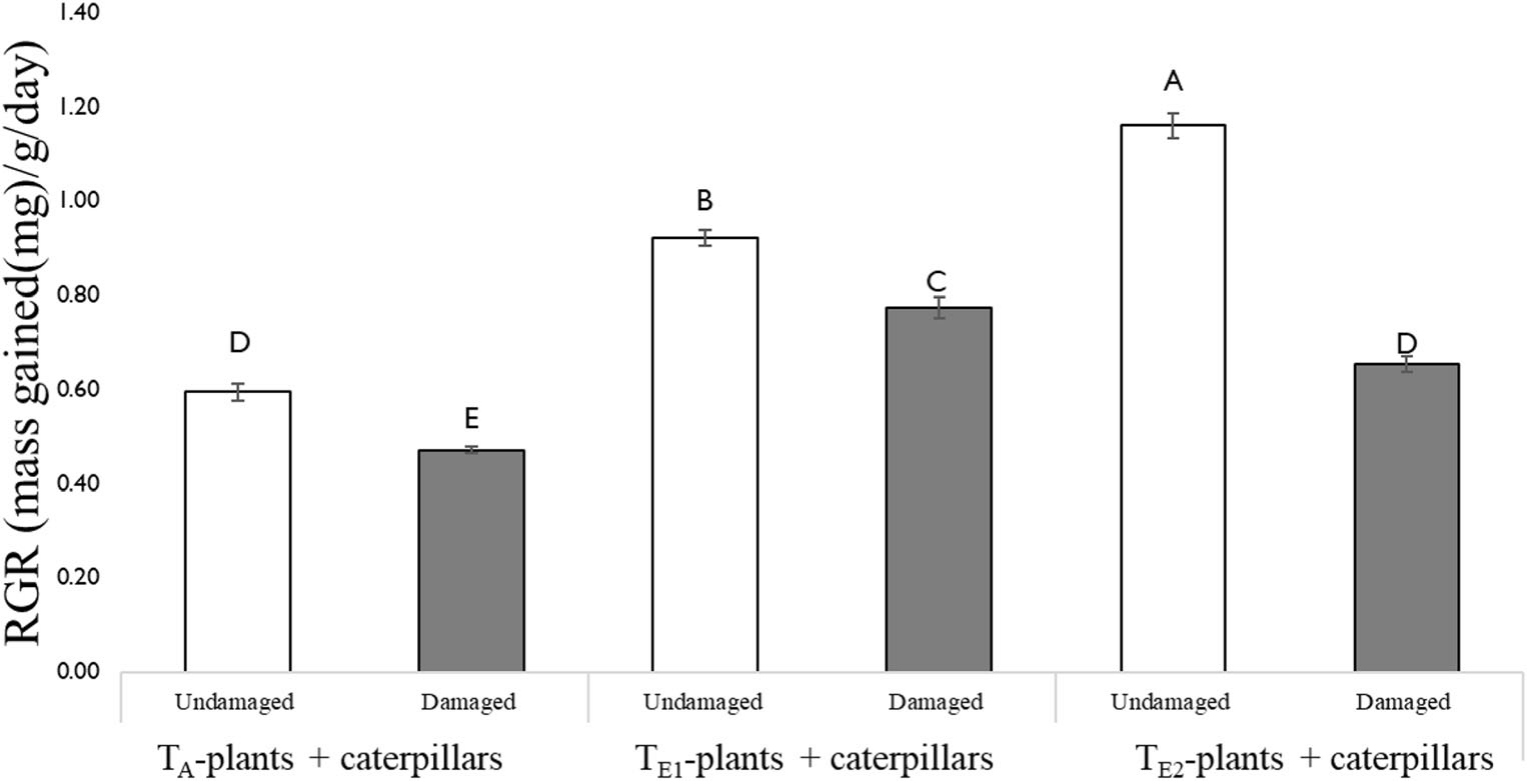
Effect of temperature on relative growth rate (RGR) (mass gained((mg)/g/day)) of 1st instar *H. zea* fed on detached leaves (damaged or undamaged) from plants grown at three different day/night temperatures: 25 °C/14 °C (ambient temperature; T_A_-plants + caterpillars), 30 °C/18 °C (elevated temperature 1; T_E1_- plants + caterpillars) and 35 °C/ 22 °C (elevated temperature 2; T_E2_- plants + caterpillars). Insects and leaves were placed in a bioassay cup and placed inside respective growth chambers during the experiment. Fifth instar *H. zea,* reared at a common day/night temperature (CT: 23 °C/19 °C) were used to damage leaves and the bioassay was conducted 48 h post-damage. Bars are mean ± SEM and means with different letters are statistically different as determined by a Tukey HSD. There was a significant independent and interactive effect of temperature and insect damage on herbivore growth (temperature: F = 161.4, df = 2, P < 0.001; insect damage: F = 188.5, df = 1, P < 0.001; temperature× insect damage: F = 43.0, df = 2, P < 0.001)

There was a significant effect of temperature and previous insect damage on herbivore growth (Fig. 8). The relative growth rate (RGR) of larvae was lowest on undamaged leaves from T_A_-plants, followed by T_E1_- and T_E2_-plants. However, the percent reduction in growth was comparatively higher on damaged leaves from T_E2_-plants. On average, RGR was reduced by 1.4-fold, 1.2-fold, and 1.16-fold on damaged leaves compared to undamaged leaves from plants grown under T_E2_, T_A_, and T_E1_ regimes, respectively.

#### Photosynthesis Rate

**Fig. 9:**
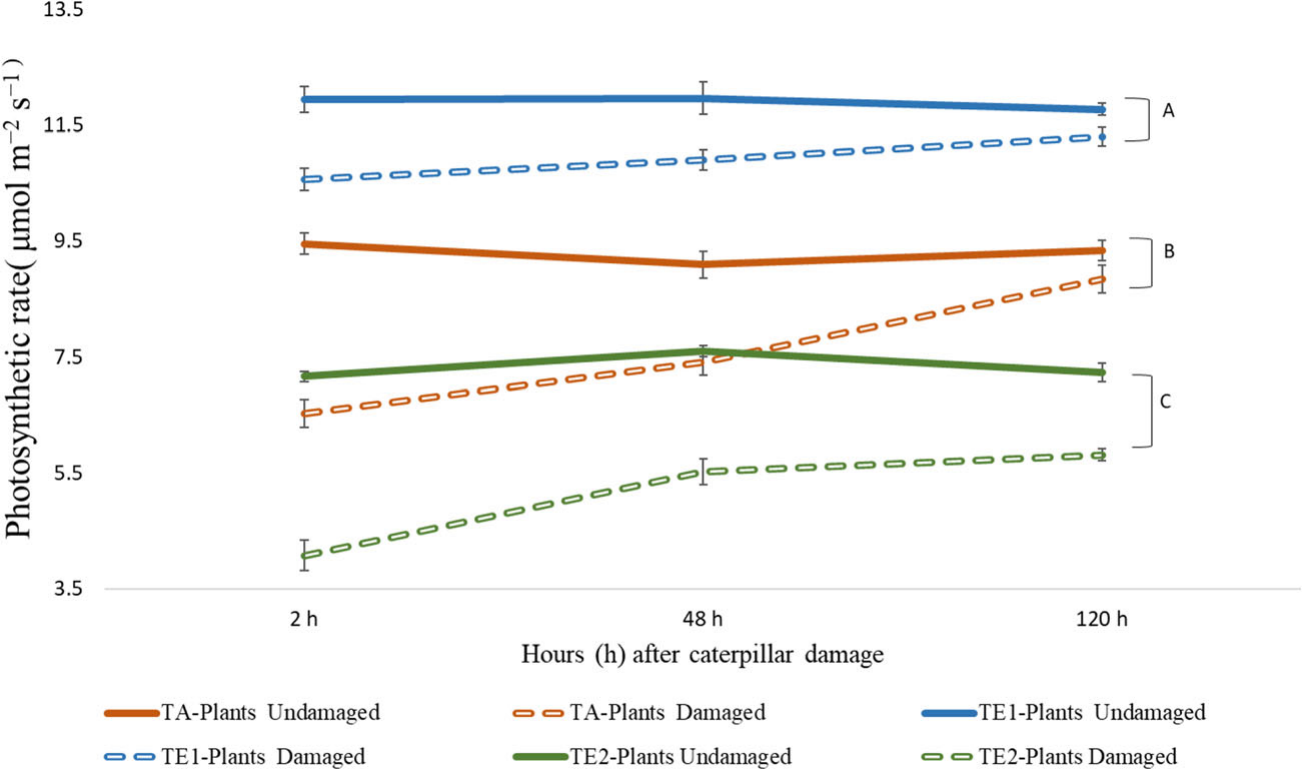
Rate of photosynthesis (μmolm^−2^ s^−1^) in undamaged (control) and damaged (by *H. zea*) leaves (treatment) at 2 h, 48 h and 120 h post-feeding periods at three different day/night temperatures: 25 °C/14 °C (ambient temperature; T_A_-plants), 30 °C/18 °C (elevated temperature 1; T_E1_-plants) and 35 °C/ 22 °C (elevated temperature 2; T_E2_-plants). Fifth instar *H. zea,* reared at a common day/night temperature (T_C_: 23 °C/19 °C), were used to damage leaves. Bars are mean ± SEM and different letters indicates a statistically difference. There was a significant effect of temperature (F = 998.9, df = 2, P < 0.001), insect damage (F = 305.6, df = 1, P < 0.001) and time (F = 20.2, df = 1, P < 0.001) on photosynthetic rates of leaves. Two-way interactive effects were also significantly different (temperature × time: F = 4.4, df = 4, *P* < 0.005; temperature × insect damage: F = 14.8, df = 2, P < 0.001; time × insect damage: F = 31.3, df = 2, P < 0.001)

Temperature and insect damage significantly affected leaves’ photosynthetic rates, and the recovery of photosynthetic capacity after herbivore damage varied with time (Fig. 9). In both damaged and undamaged control leaves, photosynthetic rate was highest in T_E1_-plants followed by T_A_- and T_E2_-plants. In damaged leaves, photosynthesis remained consistently lower compared to undamaged controls throughout the experiment, but varied greatly among temperature treatments. Post-hoc results are presented in Supporting Information Table S1.

Recovery of photosynthetic rate post herbivore damage varied with time and temperature treatments. At 2 h post insect damage, photosynthetic rate in damaged leaves was reduced by 31.0%, 11.5%, and 43.1% compared to the undamaged control leaves from T_A_-, T_E2-_ and T_E2_-plants, respectively. Within temperature treatments, photosynthesis after insect damage was most inhibited in leaves from T_E2_-plants (% reduction in photosynthetic rate- 2 h/48 h/120 h; 43.1%/27.3%/19.6%), with a drastic reduction immediately after damage; these plants failed to recover to the level of the control rate until 120 h post-damage. Photosynthetic activity of leaves from the T_A_-plants was also affected strongly by insect feeding; however, it recovered to some extent during the post-damage period (% reduction in photosynthetic rate- 2 h/48 h/120 h; 31.0%/18.5%/5.3%). Photosynthesis on leaves from T_E1_-plants was least affected 2 h after damage (11.5%) and recovered to a greater extent at 120 h post-damage (% reduction in photosynthetic rate- 48 h/120 h; 8.8%/4.0%).

#### Regrowth Ability (Compensatory Growth)

**Fig. 10:**
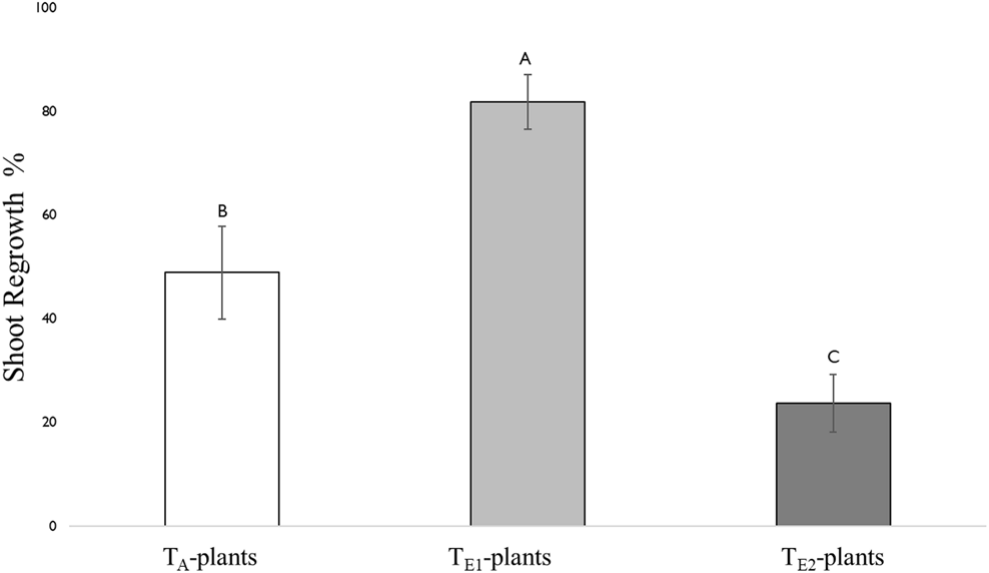
Shoot regrowth (%) of plants grown at three different day/night temperatures: 25 °C/14 °C (ambient temperature; T_A_-plants), 30 °C/18 °C (elevated temperature 1; T_E1_-plants) and 35 °C/ 22 °C (elevated temperature 2; T_E2_-plants). Bars are mean ± SEM and means with different letters are statistically different as determined by a Tukey HSD. Temperature significantly affected the rate of shoot regrowth (F = 408.38, df = 2, P < 0.001)

Temperature affected the rate of shoot regrowth (Fig. 10). On average, the regrowth percentage (%) for T_E1_-plants was 1.7-fold and 2.2-fold higher than for T_A_- and T_E2_-plants, respectively. T_E2_-plants showed considerably less capacity to compensate for shoot loss.

## Discussion

Temperature is one of the most important abiotic factors affecting both insects and plants. Temperatures are projected to increase around the globe for the foreseeable future. However, predicting the impacts of temperature change in an ecological system is complex because the response of individual species may be asymmetric. Each organism in the system may react differently to temperature change, and the interaction of species may alter responses of the individual species in an asymmetric manner. The asymmetry will produce new relationships among species, further complication predictions. In our tomato/herbivore system we found an asymmetric effect of elevated temperature on insects and plants, which consequently altered overall herbivore-plant interactions. Patterns of variation included differences in insect and plant growth, production of herbivore salivary elicitors, plant defensive protein activities and their inducibility, leaf trichome density, impacts on herbivore growth rates and plants’ tolerance ability. The effect of temperature on a plant defense elicitor, GOX, has not been previously reported.

The growth rate of an insect herbivore, *H. zea,* was accelerated with elevated temperatures when fed on an artificial diet. This is consistent with the prediction that, within physiological limits, temperature increase accelerates insect growth (Bale et al. 2002; Berggren et al. 2009). For *H. zea,* maximum and minimum temperature threshold are 12.5 °C and 36 °C respectively (Butler 1976; Mangat and Apple 1966). Higher larval weight is positively correlated with fecundity (Honěk 1993), whereas accelerated growth increases the number of generations per year, thus, reducing the window of vulnerability of the herbivore to predators and pathogens (Jaworski and Hilszczański 2013). In contrast to larval weight, pupal weight was reduced at higher temperatures (Atkinson 1994). A negative correlation between accelerated larval growth rate and pupal mass has also been demonstrated in the Monarch caterpillar (*Danaus plexippus*) (York and Oberhauser 2002) and tobacco hornworm (*Manduca sexta*) (Kingsolver 2007)*.*

Amounts of the salivary defense elicitor GOX were significantly higher in caterpillars reared at low temperatures compared to a warmer temperature. A reduced level of GOX in caterpillars reared at a warmer temperature may be a result of a tradeoff between the investment in body size and immunity at higher temperatures (Triggs and Knell 2012). Changing nutritional demand at higher temperatures may have also negatively affected the level of salivary elictor production (Hu et al. 2008; Lee et al. 2015). Interestingly, while *gox* gene expression was not significantly different among larvae grown at different temperatures, a higher level of GOX protein was observed in T_A_-caterpillars. Transcript levels (mRNA) are generally a good indicator of enzyme expression, however, there are various post-transcriptional processes (e.g., increased protein half-life) that are important to the final synthesis of a protein, which might have affected the correlation (Maier et al. 2009). While correlations between salivary defense elicitor protein levels and temperature have not been previously reported, there are studies on the effect of temperature on immune-related enzymes (Ouedraogo et al. 2003; Adamo and Lovett 2011; Perry 2017). For example, Adamo and Lovett (2011) found increased activity of two immune-related enzymes, phenoloxidase and lysosome-like enzymes in the cricket (*Gryllus texensis*) when the temperature was enhanced by 7 °C above average field temperature (26 °C). In contrast, Perry (2017) reported weakened immune functions at a warmer temperature (28.5 °C compared to 21.5 °C) in Drosophila (*Drosophila melanogaster*). It should be noted that GOX, besides its role in induction of plant defenses, also plays a role in cellular immunity (Musser et al. 2005).

Temperature influenced plant defense responses- a) by impacting temperature-sensitive plant defensive traits (plant-derived) and b) through temperature-induced changes in the ability of caterpillars to elicit plant defensive proteins (herbivory-derived). Further, the plant-derived effects varied between undamaged and damaged leaves. In undamaged leaves, constitutive defensive enzyme activities increased initially with increases in temperature, but were significantly reduced at the highest temperature regime. A similar result was reported for broccoli (*Brassica oleracea var. italica*) where seedlings grown at a comparable temperature (30/15 °C: day/night) to our experiment had significantly higher glucosinolate (GS) levels compared to those grown at lower temperatures (22/15 °C and 18/12 °C) (Pereira et al. 2002). Rivero et al. (2003) also reported a reduced level of PPO and POX activities in tomatoes at a warmer temperature of 35 °C. In contrast, in response to herbivory damage, the percent induction of defensive enzymes was highest in leaves grown at the highest temperature (T_E2_- plants). While very little information exists on the effect of elevated temperature on induction of plant defenses (Bidart-Bouzat and Imeh-Nathaniel 2008), a few studies have reported a higher induction of defensive enzymes in response to other environmental stressors. For example, there was significant induction in *Arabidopsis thaliana* of GS in response to insect feeding under drought stress and elevated C0_2_ (Bidart-Bouzat et al. 2005). Interestingly, induction of defensive proteins, in addition to the plant-derived effects, were also affected by changes in GOX in the herbivore; a low level of induced defensive proteins in plants was coupled with a reduced level of GOX in caterpillars reared at an elevated temperature (Tian et al. 2012). The overall implications of elevated temperature on plant defense responses should, therefore, reflect a composite effect of both plant and herbivory-derived effects.

When *H. zea* fed on tomato leaves, growth rate varied with temperature and was further affected by feeding on damaged versus undamaged leaves. Elevated temperatures accelerated *H. zea* growth on undamaged leaves (Gillooly et al. 2001; O’Connor et al. 2011). A similar finding was reported by Lemoine et al. (2014), where most herbivores (from 21 herbivore-plant pairs) from three orders (Lepidoptera, Coleoptera, Hymenoptera) demonstrated a higher consumption rate within a range of average temperatures of 20 °C and 30 °C. In contrast, larval growth in damaged leaves yielded a variable response; the larval growth rate increased at T_E1_ -temperature, however, it was reduced at the highest temperature regime (T_E2_). The percent reduction in larval growth rate between undamaged vs damaged leaves was also highest on leaves grown under the highest temperature regime, which indicated that T_E2_-plants elicited higher levels of resistance once attacked, which in turn, negatively influenced herbivore performance. Higher inducibility of PPO and TPI in T_E2_-plants may have contributed partly to a higher resistance against *H. zea* larvae. Previous reports have shown strong larval growth inhibition with induction of PPO and TPI (Duffey and Stout 1996; Felton et al. 1989; War et al. 2012). The variation in insects’ response to undamaged and damaged leaves illustrated the importance of induced resistance when estimating the impact of environmental changes on insect-plant interactions. Interestingly, higher leaf trichome densities at warmer temperatures failed to affect herbivore growth; therefore, trichomes in our system may play a physiological role to help plants adapt to temperature stress instead (Bickford 2016; Xiao et al. 2017). Additionally, leaf trichomes in tomatoes have been found to offer resistance mostly against small insects such as whiteflies (Bleeker et al. 2009; Firdaus et al. 2012), leafhoppers (Dellinger et al. 2006; Kaplan et al. 2009) and mites (Maluf et al. 2007).

The ability of a plant to tolerate stresses was compromised at the highest temperature regime (T_E2_) as measured by the growth, photosynthesis recovery and regrowth ability, whereas plant grown at the TE_1_ regime were the most tolerant. For tomatoes, the T_E1-_ temperature regime corresponds to an optimum temperature range for growth and production, whereas T_E2_ is above-optimum (Berggren et al. 2009; Hazra et al. 2007;). Reduced vegetative growth of plants may also affect reproductive success due to limited energy reserves (Sumesh et al. 2008). A decline in photosynthetic activity (Sharkey and Zhang 2010; Todorov et al. 2003) and regrowth ability (Han et al. 2015) with above-optimum temperatures has been previously reported. However, other reports found no evidence of compensatory photosynthesis and growth responses to herbivory and mechanical damage, respectively (Han et al. 2015; Retuerto et al. 2004; Strauss and Agrawal 1999).

**Fig. 11:**
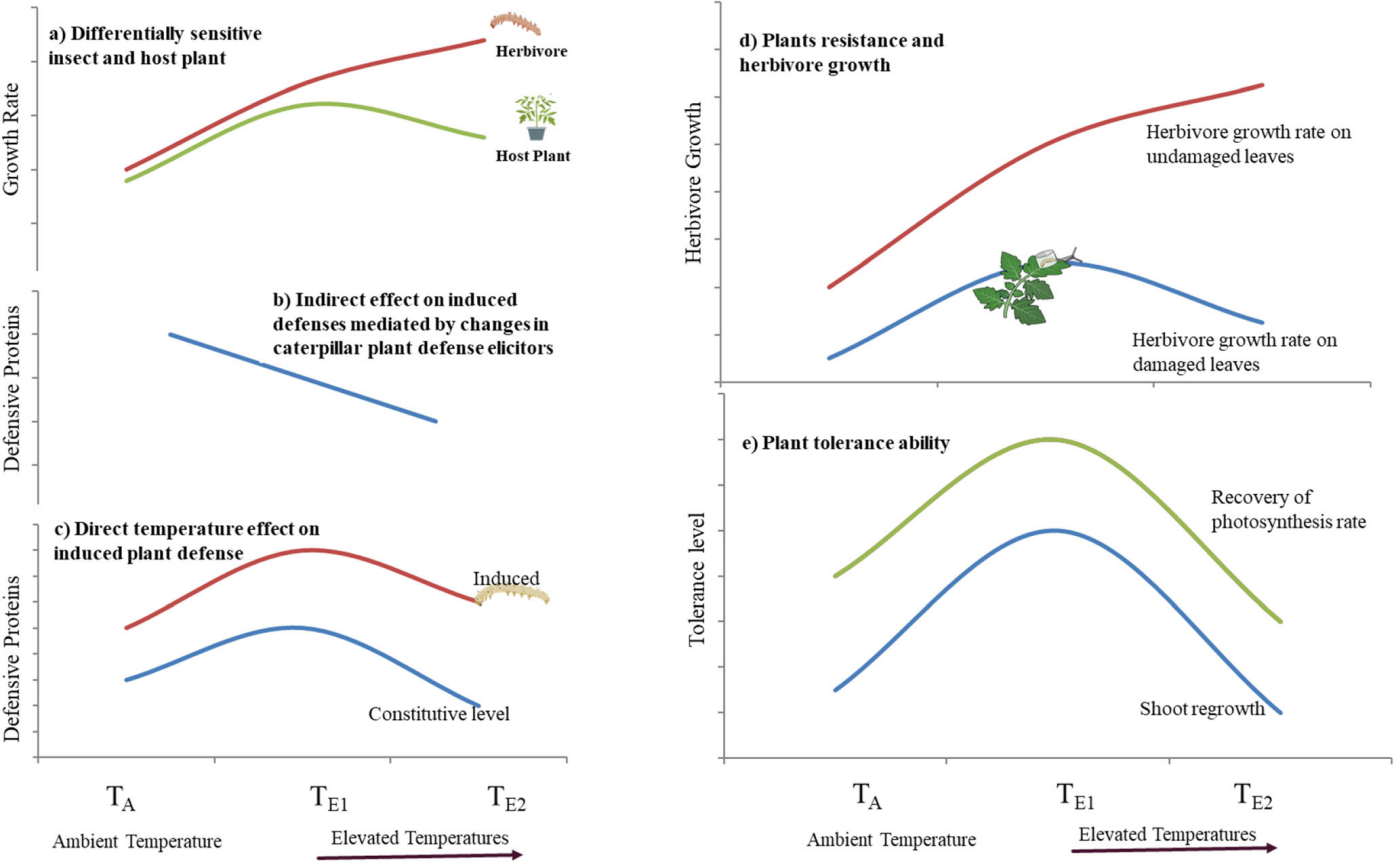
Graphical illustrations of major findings a) Within the experimental temperature range, when insects and plants were reared and grown independently, growth of insect herbivores continued to rise with temperature (red line), whereas tomato growth increased initially and declined at the highest temperature (green line) b) Temperature altered induced plant defense by influencing level of caterpillar plant defense elicitor, GOX (herbivory-derived); Activity of GOX was reduced at a warmer temperature c) Consitutive level of plant defensive proteins increased initially but declined with elevated temperature (blue line). Inducibility (induced/constitutive) of plant defensive proteins, however, was highest in plants grown at highest temperature regime d) When tomato plants were exposed simultaneously to temperature and herbivore treatment, plant resistance mechanisms were enhanced resulting in reduced herbivore growth (blue line) e) Plants’ tolerance to temperature and herbivory stress as measured by photosynthesis recovery rate and shoot regrowth increased significantly initially but declined at the highest temperature

Within the experimental temperature range, our study revealed that insects are differentially sensitive compared to their host plants based on a phenotypic response (growth); insect growth was accelerated in both T_E1_ and T_E2_-temperature regimes, whereas, plant growth was increased initially (T_E1_) but reduced significantly at T_E2_ (Fig. 11**a**). This may disrupt phenological synchrony affecting insect herbivore populations (Renner and Zohner 2018). For example, insects like Japanese beetles (*Popilia japonica*) may emerge earlier than their hosts (soybean and corn) as a result of climate warming, and therefore, have to feed on low quality foliage negatively affecting herbivore fitness (Delucia et al. 2012). Similarly, competitive relationships among herbivores may also depend on temperature, as was shown with two aphid vector species of barley yellow dwarf virus (Porras et al. 2018).

A novel finding that salivary elicitors of induced plant defenses in caterpillars is regulated by temperature is also reported here. Temperature change not only influenced the insect’s metabolic activity but also its capacity to manipulate plant defenses (Fig. 11b). Future studies are warranted to determine the adaptive ability of herbivores to respond to changes in temperature by altering the level of plant elicitors. Inducibility of plant defenses to insect herbivory was highest in plants grown at above-optimum temperatures (T_E1_) and larval growth response varied between previously damaged and undamaged leaves (Fig. 11c and d). Some of these induced effects persist over an entire season, therefore, may have a significant impact on overall crop losses (Paudel et al. 2014; Strapasson et al. 2014). This emphasizes the importance of induced resistance to estimate the impact of climatic change on insect-plant interactions (Paudel et al. 2019), which has generally been overlooked in past studies. In contrast to plants’ resistance, tolerance ability as measured by the photosynthesis recovery rate and shoot regrowth increased initially but was compromised at the above-optimum temperatures (T_E2_) (Fig. 11e).

Elevated temperature thus produced an asymmetric effect between an herbivore and its host plant, illustrating the complexity of changes in insect-plant interactions that could result as the climate warms. In theory, while activity of insect herbivores is expected to increase with global warming, independent and interactive changes in insect and plant traits as demonstrated by our results will determine the amount of crop losses. Therefore, the potential developmental plasticity of insects and plants in coping with environmental changes as well as a transformation of the interactions between them will determine species distribution and community structure. Predicitions of the future under climate change that do not take this complexity into consideration will be unconvincing.

## Electronic supplementary material

ESM 1 (DOCX 17 kb)
