# Supplementary material for: Asymmetric Responses to Climate Change: Temperature Differentially Alters Herbivore Salivary Elicitor and Host Plant Responses to Herbivory
Source: J Chem Ecol. 2020 Jul 23;46(9):891–905. doi: 10.1007/s10886-020-01201-6 (PMC7467972; doi:10.1007/s10886-020-01201-6)
Supplement: Supplementary file 1 — (DOCX 17 kb) [file 10886_2020_1201_MOESM1_ESM.docx]

**Supplementary information**

Table 1: Post-hoc Comparisons for rate of photosynthesis (μmol m^−2^ s^−1^) in undamaged and damaged (*Helicoverpa zea*) leaves during 2 h, 48 h and 120 h post-feeding periods at three different day/night temperature regimes; a) 25 °C/ 14 °C b) 30 °C/ 17 °C, and c) 35 °C/ 22 °C. Means were compared using the Tukey Method at 95% confidence. Means that do not share a letter are significantly different.

| Temperature*Time*Treatment | N | Mean | Grouping | | | | | | | | |
| --- | --- | --- | --- | --- | --- | --- | --- | --- | --- | --- | --- |
| 30°C/17°C 48 h Control | 11 | 11.9636 | A |  |  |  |  |  |  |  |  |
| 30°C/17°C 2 h Control | 11 | 11.9427 | A |  |  |  |  |  |  |  |  |
| 30°C/17°C 120 h Control | 11 | 11.7755 | A | B |  |  |  |  |  |  |  |
| 30°C/17°C 120 h Insect Damage | 11 | 11.3045 | A | B | C |  |  |  |  |  |  |
| 30°C/17°C 48 h Insect Damage | 11 | 10.9091 |  | B | C |  |  |  |  |  |  |
| 30°C/17°C 2 h Insect Damage | 11 | 10.5709 |  |  | C |  |  |  |  |  |  |
| 25°C/14°C 2 h Control | 11 | 9.4582 |  |  |  | D |  |  |  |  |  |
| 25°C/14°C 120 h Control | 11 | 9.3436 |  |  |  | D |  |  |  |  |  |
| 25°C/14°C 48 h Control | 11 | 9.1 |  |  |  | D |  |  |  |  |  |
| 25°C/14°C 120 h Insect Damage | 11 | 8.8491 |  |  |  | D |  |  |  |  |  |
| 35°C/22°C 48 h Control | 11 | 7.5973 |  |  |  |  | E |  |  |  |  |
| 25°C/14°C 48 h Insect Damage | 11 | 7.4127 |  |  |  |  | E | F |  |  |  |
| 35°C/22°C 120 h Control | 11 | 7.2345 |  |  |  |  | E | F |  |  |  |
| 35°C/22°C 2 h Control | 11 | 7.1682 |  |  |  |  | E | F |  |  |  |
| 25°C/14°C 2 h Insect Damage | 11 | 6.5291 |  |  |  |  |  | F | G |  |  |
| 35°C/22°C 120 h Insect Damage | 11 | 5.8173 |  |  |  |  |  |  | G | H |  |
| 35°C/22°C 48 h Insect Damage | 11 | 5.5191 |  |  |  |  |  |  |  | H |  |
| 35°C/22°C 2 h Insect Damage | 11 | 4.0764 |  |  |  |  |  |  |  |  | I |
